# Politeness Strategies in Permission-Asking Speech Acts: A Cross-Cultural Study of L1 and L2 English Speakers

**DOI:** 10.3390/bs16101845

**Published:** 2026-10-08

**Authors:** Hafiza Tahira, Özge Razı

**Affiliations:** English Language Teaching (ELT) Department, Faculty of Education, Cyprus International University (CIU), Mersin 10, Nicosia 9258, North Cyprus, Turkey

**Keywords:** permission speech acts, pragmatic variation, cross-cultural pragmatics, politeness, EFL learners, sociocultural awareness

## Abstract

In interlanguage pragmatics, effective communication enhances pragmatic awareness and mutual understanding, while the art of asking permission enables the speaker to convey clear information, allowing the listeners to comprehend, respond and decide. This cross-cultural study investigates politeness strategies of asking permission (PSAs) utilized by native (British) and EFL learners from Turkish, Congolese and Moroccan backgrounds. A qualitative content analysis of (504) responses was employed to elicit the responses of all participants to identify developmental strategy patterns across all groups. The findings revealed a significant distinction between L1 (native British) and L2 (EFL learners) speakers of English: L1 speakers employed higher frequencies of negative politeness strategies as compared to other groups in all contexts (power, distance and imposition); EFL learners showed variations in the production of permission-asking speech acts (PSAs). They frequently used positive politeness strategies featuring solidarity markers and increased negative politeness use, suggesting contextual awareness across all groups while seeking permission in higher-status settings. Both L1 speakers and EFL learners demonstrate concern for social harmony and face-saving; they employ culturally distinctive strategies to achieve these shared communicative goals. This pattern reflects culturally embedded pragmatic variations by shaping learners’ choices based on L1 cultural nuances. This exploratory study contributes to cross-cultural pragmatics research by highlighting PSA production patterns across diverse learner populations and implicating intercultural training and providing empirical insights to inform culturally aware pedagogy in educational settings.

## 1. Introduction

Effective communication and pragmatic ability are pivotal skills that allow learners to convey meaningful and clear messages to fulfill the aspects of conversation (Austin, 1962; Searle, 1969). This conversation is a source of human interaction; it does not just exchange information but also performs actions through words (Searle, 1969). This issue, however, is rather difficult for second language learners. Producing socially accepted and appropriate conversations challenges the L2 learners (Purpura, 2004; Bachman & Palmer, 2010). The speech acts in their nature differ from culture to culture and are utilized as a tool to apply cultural norms. This variation in language and cultures creates challenges for L2 learners in their production of speech acts, as it requires them to consider the task’s demand, L1 background, and the need to encourage social harmony (Taguchi, 2007a).

Searle’s (1969) study on speech acts is considered fundamental to the functions of language across cultures and languages, providing a systematic framework for understanding how speakers accomplish pragmatic goals and produce speech acts, e.g., permissions, directive speech acts and requests through their choice. To explore these choice researchers investigated EFL learners’ capabilities of production and suggested pedagogical implications to mitigate these challenges according to their needs (Hudson, 2001; Taguchi, 2007b; Youn, 2019). When conversing, L2 learners should take sociocultural appropriateness and context-sensitive communication into consideration, as they are important elements of socially acceptable interactions (Ross & Kasper, 2013; Ren, 2022). Brown and Levinson (1987) proposed the politeness theory to explain how speakers interact in society and establish social relations utilizing various interpersonal strategies (bald on-record, positive politeness, negative politeness, and off-record) to save their “face” and maintain a person’s public image.

Brown and Levinson’s (1987) politeness theory is a fundamental concept in pragmatics, presenting a social phenomenon of four strategies of face-threatening acts in communication. The first strategy, bald on-record, presents directness in the utterances of the interlocutors when the speaker possesses a strong sense of authority over the listener (Brown & Levinson, 1987). The second strategy, positive politeness, addresses the hearers’ positive face, their desire for approval, solidarity, acceptance, and inclusion in-group terms (Brown & Levinson, 1987; Yus, 2011). The third strategy, negative politeness, focuses on the hearer’s negative face, their desire to be autonomous, freedom from imposition and maintaining a safe distance from the interlocutor. It is presented through hedges, apologies, distancing devices and indirectness (Brown & Levinson, 1987; Culpeper & Demmen, 2011). The fourth strategy, off-record, utilizes implicature and ambiguity to ensure that the communicative aim is deniable, hence assigning interpretive responsibility to the listener (Brown & Levinson, 1987; Thomas, 1995). These strategies are chosen based on social factors; relative power of the interlocutors (P), social distance (D) and rank of imposition in face-threatening acts. This framework is widely used to examine how to pick up and use these strategies in various power and distance circumstances (G. Kasper & Rose, 2002; Taguchi, 2006). G. Kasper and Blum-Kulka (1993), Moyer (2013), Spillner (2017), and Swan (2001) discussed how L2 users tackle grammar structure, pronunciation, and vocabulary and produce speech acts in the target language differently from L1 users. Overlooking these pragma-linguistics and intercultural variations leads to misunderstandings, rudeness, and miscommunication (Alisoy, 2025). Therefore, teaching pragmatic skills and intercultural awareness is vital for fostering learners’ interactional effectiveness.

In sociolinguistic interactions, permission speech acts and politeness play a pivotal role, particularly in the production of second language utterances (Jacobson, 1976). The concept of permission entails dual interpretations: denotatively, it implies allowing the speaker to do (act) something (Moore, 2004), and pragmatically, it functions as an inquiry about an individual’s capability to perform an action. Research on speech acts of permission is expanding within the field of pragmatics, and politeness is a factual element. It involves consent in requesting as well as avoiding the intensity of imposition between the speakers (Alzeebaree, 2021). Brown and Levinson (1987) defined face-threatening acts (FTAs) as actions that can negatively affect an individual’s reputation or self-image. These actions affect both the listener’s and the speaker’s positive or negative face. To mitigate potential harm to either party’s face, the speaker must employ politeness strategies (Leung et al., 2008; Yang, 2026). Pragmatically, inadequate or incorrect language use can undermine the interaction’s objective and cause discomfort between speakers.

A request for permission is considered the most indirect and face-threatening speech act (Blum-Kulka & Olshtain, 1984). In various cultures, clarity and indirectness are valued, and individuals often employ different strategies to ask for permission to avoid confrontations and misunderstandings based on their cultural norms (Aziz, 2002; Berrios et al., 2025). Abdul-Ameer and Shaymaa (2014) investigated Iraqi EFL learners’ performance of speech acts and discovered the most commonly utilized strategies of politeness. To maintain positive social relations and harmony, speech acts of permission are frequently employed in educational settings (Hanna & Richards, 2019). To produce these particular acts (permission), it is mandatory for learners to pay close attention to various social norms and consider power dynamics to communicate effectively and accurately when having a dialog with a teacher vs. a peer (G. Mammadova, 2025; T. Mammadova, 2025; Ji et al., 2025). Although several studies (e.g., Derakhshan & Shakki, 2021, 2023; Kostromitina, 2024; Mashhadi & Memari, 2025) extensively investigated L2 pragmatic request speech acts in relation to learners’ L1 background, a few studies on the sub-act of asking permission (Alzeebaree, 2021; Tran, 2022; Al-Ghazo, 2024; Rafiq & Yavuz, 2024) have concentrated on permission speech acts (PSA); notable exceptions include Alzeebaree’s (2021) and Tran’s (2022) cross-cultural study on PSAs and Naiditch and Ebsworth’s (2006) comparative analysis of L1 Americans and L2 Brazilian Portuguese speakers’ permission request. Alzeebaree’s (2021) research on the realization of permission speech acts examined how EFL learners manage to produce, understand and realize these acts. Al-Ghazo (2024) investigated the force and power of language expression by asking Jordanian EFL learners to identify permission speech acts, and most of them were able to identify these expressions.

English as a second language (EFL) learners demonstrate various uses of speech act strategies when they belong to distinct linguistic and cultural backgrounds. These variations result from rooted L1 cultural backgrounds; cultural values might influence the way people behave and social interactions (Hofstede, 2001). Research on speech acts suggested that Turkish speakers’ use of direct strategies and less use of negative politeness strategies were influenced by L1 transfer, and their choices varied compared to English speakers (Maftuna & Usenmez, 2026). Özcan (2022) revealed that minimal use of softening devices, hedging and apology markers was evident in L1 pragmatic transfer Turkish communication, which is mainly oriented toward directness and information efficiency, rooted in cultural dimensions and L1 transfer (Şanal & Ortaçtepe, 2019). Arabic cultural values are reflected in Talay’s (2022) study, where Moroccans’ English language speakers’ strategies were influenced by falling back on their native pragmatic norms; they also employed solidarity and warm relationship-preferred politeness strategies (Alaoui, 2011; Abidi, 2022; Ichkhakh, 1987). Ed-Deraouy and Sana (2024) claimed that Moroccan EFL learners exhibit a nuanced understanding of sociolinguistic variables; they are not merely passive recipients of linguistic norms but actively adapt their linguistic choices based on the social and relational contexts. Bouknify (2025) demonstrated that Moroccan learners exhibit pragmatic competence through task-based assessment. Lastly, recent research demonstrates that Congolese multilingualism shapes pragmatic competence across languages. Tsoumou (2019) revealed that Congolese speakers used politeness markers and in-group communication to preserve social relationships. These findings demonstrate that Congolese pragmatic competence involves sophisticated management of politeness across multiple languages. Congolese speakers reflect cultural communication norms toward relationship maintenance, and their collective identity reflects L1 pragmatic patterns (Ntouta, 2024). These EFL learners from various linguistic and cultural backgrounds exhibit a diverse but coherent pragmatic context which reflects the dominance of L1 linguistic transfer that demonstrates various patterns in their strategy choices rather than pragmatic failure.

The novelty of the present study does not reside in a single discovery; it comprises several contributions, including a focus on underexplored speech acts (permission-asking) within interlanguage pragmatics (IPL), inclusion of linguistically and culturally diverse learner populations (Congolese learners who have received limited attention), a theoretically stimulating finding that L2 learners reflected harmony with L1 speakers in higher-status contexts, and, lastly, its integration of socio-pragmatic awareness as an explanatory variable for learners’ strategy production choices.

### Current Study

This research examines British English speakers’ (L1) and EFL learners’ (L2) politeness strategies in the production of asking permission speech acts in various university settings. EFL learners are Turkish, Congolese and Moroccan EFL learners who participated in the study. The rationale for focusing on these groups is the availability of enrolled first-year students, which allows the researcher to conduct the study for a diverse and reachable sample. The research seeks to elucidate how English speakers and EFL learners produce permission speech acts, their strategies, and the developmental variations in the given contexts and socio-pragmatic norms. It highlights the cultural differences in politeness, face-saving acts and social harmony, which are crucial in cross-cultural communications. The study contributes to a culturally aware method that can help to avoid misunderstandings among the speakers due to various cultural and social differences such as classroom settings, academic settings, and professional interactions. The findings of this research demonstrate how cultural norms affect the choices of strategies while producing permission speech acts, emphasizing the significance of cultural awareness in teaching pragmatic techniques and assessment practices. Diverse cultural backgrounds can influence the pragmatic choices of speakers. Native speakers (L1) manage to keep the balance, and EFL learners (L2) show variations in developmental patterns in different contexts. These variations highlight the gaps in learners’ socio-pragmatic awareness and demonstrate the effect of cultural background on permission-asking settings (Alzeebaree, 2021).

Consequently, this research aims to discover whether EFL learners’ strategy choices align with those of the L1 speakers of English. Ultimately, this study aims to promote respectful and effective interactions in multicultural settings through two research questions. 1. What politeness strategies does each group from British, Turkish, Congolese and Moroccan backgrounds employ? 2. Do these strategy choices differ across socio-pragmatic contexts (to an equal status, to a higher status, to a stranger)?

## 2. Literature Review

Successful and appropriate communication requires pragmatic ability to comprehend the given information. It helps to reduce misunderstandings among the interlocutors and contributes to the components of second language acquisition within communicative competence (Farashaiyan & Muthusamy, 2017; Tanck, 2004; Canale, 1983; Bachman & Palmer, 1996; Yule, 1996; Crystal, 2001; Leech, 1990). To enhance the effectiveness of communication, understanding of cultural nuances is a crucial aspect of pragmatic competence. In a cross-cultural study, Usmani and Almashham (2024) emphasized that to achieve effective and appropriate interaction, it is vital to attain awareness of cultural differences and social norms. According to Ichkhakh (1987) and Abdou (1999), research on cross-cultural pragmatics remained limited within the permission framework. The concept of culture itself poses significant challenges for researchers, and it encompasses individuals’ demeanor, norms, and social events: it directly influences their actions across contexts and is transmitted through generations by humans within a community (Williams, 1983; Hall, 1990).

Studies highlight valuable insights into the role of language in social norms and various contexts by providing a theoretical framework (Austin, 1962; Revita, 2008; Searle & Vanderveken, 1985). G. Kasper (1990) and Kranich et al. (2021) emphasized the importance of sociocultural communication and its effects on cultural diversity, politeness, indirectness and social awareness in the production of speech acts. 

### 2.1. Pragmatic Competence

A successful interaction among speakers is the ability to produce language properly and effectively, considering cultural and social contexts, which is indispensable for the production of a language (Ren, 2022). Thomas (1984) defined pragmatic competence as the skill to achieve a particular aim to understand language in various contexts of society. Hymes (1972) illustrated the importance of communicative competence, which cannot be achieved by focusing solely on grammatical knowledge, as well as enhancing skills of using linguistic components and their effective application according to the contexts of real-life situations.

These complexities emphasize the importance of enhancing pragmatic competence in EFL learners. Scholars such as Blum-Kulka (1990), Economidou-Kogetsidis et al. (2021), and Eslamirasekh (1992) conducted studies on pragmatic competence to evaluate whether EFL learners’ L2 speech acts were contextually appropriate. These studies are inspired by the twofold nature of permission speech acts, particularly examining research on diverse cultures and languages. Salih and Kurt (2025) revealed the importance of pedagogical implications in his study of speech acts that Kurdish EFL students produced pragmatically and structurally appropriate responses on post-tests, a result supported by a learner-centered approach. Furthermore, Jalilifar (2009) examined the directness levels of Australian native speakers and Iranian EFL learners using a WDCT and revealed that higher-proficiency Iranian learners overuse indirect strategies, while lower-proficiency learners favored direct ones; native speakers demonstrated more balanced strategies as compared to FEL. Ahangari and Shoghli (2011) observed that both Iranian learners and Canadians primarily utilized conventionally indirect strategies. Myrset (2022) tested learners’ request strategies using a video-prompted oral discourse completion test, revealing that clear instructions were highly effective for long-term retention of pragma-linguistic materials.

#### Pragmatic Failure

Pragmatic failure is considered a serious issue in second language communication, which occurs when learners employ linguistically appropriate utterances that are pragmatically ineffective in their target language contexts, leading to misunderstandings and misconceptions among the speakers (Salih & Kurt, 2025). Thomas (1984) presented the cross-cultural pragmatic failure theory, which was built on Leech’s (1983) distinction to formulate this failure, and divided it into two categories: pragma-linguistic failure (when speakers choose a different linguistic form and try to convey a message through direct translation of their L1) and socio-pragmatic failure (when speakers comprehend the meaning but fail to judge the social contexts). However, numerous EFL learners struggle to communicate effectively in the English language; due to this endeavor, they tend to use the tactics of direct L1 translation and cultural practices in their native languages, whether consciously or unconsciously (Amna, 2018). Brown and Levinson (1987) discovered that factorial variables such as relative power, social distance, and the intensity of imposition could influence and threaten the face of the speakers in cross-cultural communication and cause cultural complexities.

Studies have evidenced that even highly proficient second language (L2) learners may struggle to engage in the production of effective interaction in English as a second language (ESL), mainly with respect to speech acts (Economidou-Kogetsidis, 2002, 2011; Economidou-Kogetsidis et al., 2021; Blum-Kulka & Olshtain, 1984; Trosborg, 1995). Derakhshan and Arabmofrad (2018) utilized a discourse completion task (DCT) to assess Iranian EFL learners’ speech acts of requests, apologies, refusals, and thanks, revealing pedagogical implications that a consciousness-raising strategy can improve pragmatic competence. Ashoorpour et al. (2014) explored Iranian EFL pre-intermediate and advanced learners’ pragmatic and grammatical ability by utilizing DCT for request speech acts, and the findings revealed a significant difference; advanced learners outperformed in both areas.

Furthermore, Jalilifar (2009) examined the directness levels of Australian native speakers and Iranian EFL learners using a WDCT, revealing that higher-proficiency Iranian learners overuse indirect strategies, while lower-proficiency learners favored direct ones; native speakers demonstrated more balanced strategies as compared to FEL. Consequently, this failure can be overcome by pragmatic training to enhance learners’ pragmatic communication skills in appropriate intercultural settings (Rafiq & Yavuz, 2024).

### 2.2. Politeness

It is crucial to recognize that comprehension and implementation of politeness strategies may vary and differ from culture to culture, fostering productive and effective respectful communicative engagement (Holmes & Wilson, 2022). According to Islentyeva et al. (2023), the linguistic marker ‘please’ is believed to be used by the speakers to reduce the intensity of disagreement between the speakers and soften the conversation.

Politeness is considered a preliminary subject in education, vital for maintaining courtesy and dignity in the classroom for both pupils and educators. Lakoff (1974) suggested being polite in a conversation and highlighted the importance of considering others’ feelings while using language functions (speech acts) between the interlocutors; this tactic can help to avoid conflicts and misunderstandings. Tanduk (2023) stated that a lack of politeness awareness in conversations between speakers can lead to misunderstandings and confrontations; such behavior is frequently perceived as impolite. Haugh (2024) distinguished a fine line between politeness and impoliteness and considered impoliteness-as-object, which encompasses a complex set of first-order (evolves into broader societal commentary), second-order (explicit lay judgments about an interaction after it occurs), and third-order (structural analysis of the underlying social norms) assessments that are related yet conceptually distinct.

While conventional studies on politeness theories (e.g., Brown & Levinson, 1987; Leech, 1983) establish a systematic universal framework to analyze politeness, these approaches inadequately account for cultural differences and contextual factors that mainly influenced interlocutors’ production choices. This debate has particular significance for understanding permission speech acts (PSAs). On the contrary, the argumentative perspective on politeness, articulated by theorists such as Mills (2003, 2011), Watts (2003), Spencer-Oatey (2000), Haugh (2011), and Culpeper (2010, 2011), enhances the concept by underscoring context sensitivity and cultural differences. A key component to this enhancement is the distinction between politenesses 1 (routine social interaction with real conversation) and politeness 2 (theoretical conceptualized and rule-governed phenomenon), as expressed by Eelen (2001) and reformulated by Watts (2003) as first- and second-order politeness. Mills (2011) highlights that the discursive approach challenges all ideas and evaluations and is doubtful of any attempts at grand narrative or metanarrative, consequently eliminating universal politeness strategies in favor of examining how permission speech acts are dynamically constructed, interpreted and evaluated in legitimate intercultural interactions. This conceptual framework is essential for examining PSAs among diverse EFL learner populations; it acknowledges that PSAs cannot be properly evaluated through conceptual politeness theories but instead require analysis grounded in legitimate discourse, participants’ perceptions, and cultural pragmatic standards that shape suitable permission-asking patterns. Evizariza (2024) and Basis (2024) indicated that politeness can be reflected through various communication expressions such as addressing someone by their title, listening attentively, and showing respectful expressions and gestures. Ultimately, politeness is deeply linked to established social norms, demonstrating appropriate demeanors across various cultures (Zhou & Zhang, 2018). Overlooking these social and cultural norms in everyday conversations can lead to confrontations and misunderstandings among the speakers.

### 2.3. Permission Speech Acts (PSAs)

In everyday conversations, asking for permission is a fundamental aspect of receiving consent from the interlocutor. It plays a significant role in maintaining relationships, showing politeness and social harmony in various cultural contexts.

Examining permission speech acts in intercultural pragmatics requires a sophisticated understanding of theoretical and practical application of politeness in academic research. Haugh et al. (2013) examined politeness strategies in professional interactions, and the findings suggested that the speakers used hedging techniques and mitigation strategies to reduce face threats. Research on cross-cultural pragmatics reveals important patterns. Zhang (2019) revealed that Chinese speakers were more likely to utilize indirect speech act strategies as compared to English speakers. Ishihara and Cohen (2010) conducted research involving Japanese and English speakers, revealing that ineffective communication can result in misunderstandings and misinterpretations among the speakers. Overall, speech acts performed with permission are a form of illocutionary act that serves a specific purpose in communication. In these interactions, both the speaker and the listener engage in a dialog in which permission is either accepted or rejected (Ishihara & Lee, 2021).

In cross-cultural research, Blum-Kulka (1993) highlighted the strategies employed by Hebrew speakers and Native Americans for making requests, illustrating how cultural norms shape learners’ choices. Rachman (2022) studied the role of requests in Indonesian and Japanese cultures, finding that permission is considered the politest mode of communication across these societies. Betti et al. (2018) examined multiple types of speech acts in the study and discovered that learners’ skills of recognition and production were enhanced after treatment. Another cross-cultural study comprising speech act strategies addressed by Tran (2022) indicates that Vietnamese speakers use direct strategies; however, they use more politeness markers when addressing elderly people than peers, whereas English speakers demonstrated balance, utilizing conventionally indirect strategies and politeness markers across speakers.

Moreover, Aliakbari and Gheitasi (2014) examined Iranian learners’ politeness, appropriateness, formality, and their awareness in the production of permission acts. The results show that participants used highly formal, polite, and indirect language due to spending some time in an English-speaking country, which indicates that exposure to the target language culture helps to enhance language awareness and knowledge. Bardovi-Harlig (2012) noted that native speakers of English adjust their degree of formality in the use of strategies while producing permission speech acts based on context and situational standards. Conclusively, all these studies shed light on the impacts of EFL learners’ challenges and complexities in dealing with the phenomenon of social and cultural norms of L2 pragmatic competence while mitigating speech acts. 

To conclude, this pragmatic study shows that the effective use of permission speech acts (PSAs) requires both socio-pragmatic awareness (appropriate use of politeness when needed) and pragma-linguistic knowledge (understanding the forms of politeness realization) (Thomas, 1984; Ren, 2022). Research shows that speakers of English (L1 & L2) employed various types of politeness strategies (bald on-record, positive politeness, negative politeness and off-record) in different social contexts depending on power dynamics, social distance, and rank of imposition (Brown & Levinson, 1987; Culpeper & Demmen, 2011), with substantial variations due to L1 cultural background, which influences their strategy choices (Blum-Kulka & Olshtain, 1984; Tran, 2022). The literature reveals several important gaps. Previous research examined speech acts of various populations (Kurdish, Japanese, Chinese, Iranian, American, etc.); however, the combination of these three linguistically and culturally distinctive populations remains substantially understudied. The comparative perspective of this study highlights how different L1 backgrounds can influence permission strategy choices of the speakers in various contexts. Overall, these studies collectively highlight the complexities and challenges of permission speech acts and their impacts on language learning and social and cultural interactions.

## 3. Research Design

This study adopts a qualitative content analysis design with quantitative descriptive reporting to investigate how English as a Foreign Language (EFL) learners and native British English speakers realize permission-seeking speech acts in university contexts. Qualitative approaches are particularly suited to examining social and linguistic behavior because they allow researchers to explore participants’ perceptions, beliefs, and interpretations of communicative situations in depth (Moser & Korstjens, 2017; Minichiello, 1990; Creswell, 2003). Within this framework, the study focuses mainly on the politeness strategy choices made by the native and non-native speakers of English and how their pragmatic competence is influenced by social variables (power, relation, and imposition). To elicit data, the study employed the written discourse completion task (WDCT). It is widely used in the field of interlanguage pragmatic research to elicit speech acts due to its nature of enlightenment. Even though WDCTs do not provide spontaneous insights into interaction, they allow the researcher to gain a deep comprehension of participants’ responses regarding politeness, awareness of contextual settings, and preferred linguistic choices (G. Kasper & Dahl, 1991). The employed WDCT in this research was designed to achieve participants’ mediation of power, social distance, and rank of imposition when asking for permission.

### 3.1. Situational Variables

The scenarios comprised eight asking permission scenarios representing various contextual variables. These variables were adopted from Brown and Levinson’s (1987) work on face-threatening acts (FTAs). This socio-pragmatic framework includes three variables: relative power (P) between the speakers (student to professor), degree of social distance (D) to a stranger or close friend, and the rank of imposition (R), borrowing something. 

### 3.2. Research Instrument

The first version of the research instrument was developed by the researcher and fellow researchers in the field collaboratively to examine and select the appropriate scenarios of permission-based situations. Initially, the instrument consisted of fifteen scenarios; to assess the clarity and linguistic appropriateness, it was piloted on 40 EFL learners and native speakers. Based on feedback from learners and fellow researchers, seven situations were eliminated due to inconsistency and limited relevance to university contexts. The final version of the instrument contains eight situations, each representing the power relations, social distance, and the rank of imposition. To ensure the validity and reliability, three specialized professors from the pragmatics field reviewed and approved the finalized version of the instrument.

The strong inter-rater reliability was estimated in the study with the value r = 0.89 calculated using Cohen’s Kappa (κ = 0.82) for categorical coding, and Intraclass Correlation Coefficient (r = 0.89) was used for ordinal coding of politeness strategies. Both values exceed the threshold of 0.70, which indicates strong agreement. Gwet (2001) suggested that the strong agreement could be assessed through the value of ‘r,’ which indicates the closest agreement among the raters’ analysis of the data.

Table 1 represents the situational variables and scenarios. These permission scenarios consist of three variables P, D and R, which allow the researchers to examine and evaluate the responses of all the participants according to their cultures and the languages they speak.

To establish a clear and transparent coding, the study utilized a detailed coding taxonomy derived from Brown and Levinson’s framework with four politeness strategies extended with subcategories and tactical linguistic devices.

Table 2 presents coding taxonomy derived from B&L politeness strategies, sub-strategies, tactical devices and linguistic examples.

### 3.3. Participants and Data Collection

Data were collected through convenience sampling, a practical approach commonly used in exploratory qualitative research. While convenience sampling does not aim for statistical generalizability, it enables access to participants who are available, willing, and relevant to the research focus (Golzar et al., 2022; Subedi, 2023).

A total of 63 participants took part in this study, including nine British (L1 speakers of English) and 54 first-year university students (L2 speakers of English) aged 18 to 26, recruited from accessible classrooms. English as a second language speakers (EFL learners) represented diverse linguistic and cultural L1 backgrounds, including 16 Turkish (M = 8, F = 8), 19 Congolese, (M = 10, F = 9), and 19 Moroccan, (M = 9, F = 10), all of whom were enrolled in undergraduate programs at a private university in TRNC, available, and willing to participate in the study. They had studied English for varying durations (Table 3).

To ensure that the native speakers represent the target native English variety, nine British English (L1) speakers (5 female and 4 male) who were born and acquired English from birth, had spent their formative years in an English-speaking country and completed their education in the UK, and are currently working as professionals at the institution were included as a comparison group to provide contextual understanding for EFL learners’ politeness strategies. They also had spent 2–3 prolonged years in a non-native English-speaking environment.

Data were collected between 15 April and 30 May 2024. All participants completed a demographic questionnaire followed by the written discourse completion task (WDCT). Participants were assured of anonymity and informed that their data would be used for research purposes only. Participants responded to each scenario by reading and imagining themselves in the described situation and writing down what they would say to ask for permission. 

### 3.4. Analytical Approach, Coding Taxonomy and Procedure

The study employed a qualitative content analysis approach grounded in Brown and Levinson’s (1987) politeness theory; descriptive frequency analysis offers clarity regarding the use of strategies across groups (Moser & Korstjens, 2017; Creswell, 2003). This dual methodology strengthens theoretical systematic categorization of WDCT responses while remaining sensitive to contextual nuance. All data were anonymized, coded numerically, and securely stored. A deductive framework based on the four politeness strategies, bald on-record (BOR), positive politeness (PP), negative politeness (NP), and off-record (OR), guided the analysis, supplemented by inductive observations such as mitigation devices and modal patterns. This framework has been used in pragmatic studies and extensively validated in cross-cultural research; it is suitable for investigating permission speech acts (PSA). To ensure transparency and consistent coding, the study used a comprehensible coding taxonomy constructed from Brown and Levinson’s four strategies, enhanced with clearly defined sub-strategies and tactical linguistic devices.

The procedure unfolded in two stages: L1 English speakers’ responses were analyzed first to establish baseline pragmatic norms, followed by the EFL learner data, which were coded using the same framework and compared against native speakers to identify divergences, strategy overuse or underuse, L1 transfer, and developmental patterns in pragmatic competence. To ensure coding reliability and inter-rater agreement, the study employed a validation procedure. Two raters with expertise in the field who acknowledged Brown and Levinson’s framework categorized the responses into four main strategies and sub-strategies using the taxonomy presented in Table 2. All 504 written discourse completion task (WDCT) responses were independently coded by the raters using the politeness strategy coding taxonomy.

The high agreement between the raters was confirmed through Cohen’s Kappa Coefficient, and values and discrepancies were resolved through discussion. Situations where disagreements appeared typically involved multiple strategies such as: apologies, markers, and reasons; these issues were resolved through discussion and reference to the priority taxonomy, as shown in Table 2. Ethical standards were upheld throughout, with informed consent, anonymity, and secure data handling ensured.

## 4. Results

This section presents the findings of the written discourse completion task (WDCT), which examined how native speakers and EFL learners from Turkish, Congolese, and Moroccan backgrounds produced permission speech acts (PSAs) across a range of socio-pragmatic contexts. The analysis focuses on three variables central to Brown and Levinson’s (1987) politeness framework: power (P), social distance (D), and rank of imposition (R). The findings are organized thematically and supported with representative examples.

The results show how native and non-native speakers tend to produce requests for permission in various contexts.

### 4.1. Overview of Emerging Themes

Across all groups, several recurring themes were identified: solidarity and inclusivity, conventional indirectness, reason + permission OR permission + reason structures, attention getters, apologetic openers, justifications, social busyness markers, and excuses for disruption.

These themes reflect how participants mitigated face-threatening acts and adapted their strategies to different social contexts.

### 4.2. Participants’ Strategy Preference

The analysis of data from the responses (WDCT) of the groups focuses on individual evaluations of the strategies employed by British and EFL learners in using permission speech acts (PSAs). These strategies are categorized into three key variables: relative power (P=), distance (D+), and rank of imposition (R+). The framework is specifically designed to examine how permission is requested, with strategies further divided into relative categories for comprehensive analysis, each containing sub-classifications. As a result, it has been observed that learners tend to produce permission speech acts.

Data were elicited using coding and theme development methods. Analysis of the L1 and L2 speakers’ responses revealed that British speakers consistently prioritize the preservation of autonomy in their interlocutors through the use of specific linguistic markers, such as *“Do you mind,” “Would you mind,” and “Would it be ok if I…?”* When communicating with individuals of higher status, such as bosses and lecturers, there is a notable preference for negative politeness strategies. In contrast, when interacting with peers or friends, L1 speakers tend to adopt a slight use of positive politeness strategies, maintaining formality while still preserving autonomy through markers such as *“would” and “Do you mind?”* Furthermore, in conversations with strangers, British speakers often utilize apologetic markers *such as sorry to bother you…, sorry to disturb you…, excuse me*, etc. and attention getters (excuse me…) to facilitate communication. This varying use of politeness strategies reflects a nuanced approach to interpersonal interactions among L1 speakers, tailored to the status and familiarity of their conversational partners.

Table 4 presents the overall distribution of politeness strategies.

All 504 WDCT responses were coded and analyzed. Overall, politeness strategies are presented in Table 2 for all groups (L1 speakers and EFL learners). To assure the transparency of elicited data through qualitative coding taxonomy, a descriptive frequency analysis was executed for the main strategies. According to the results, it is clear that L1 speakers of English (British) employed the highest frequency of negative politeness strategies (81.9%) among the groups and a slight use of positive politeness strategies (16.7%). On the other hand, Congolese learners used the least number of negative politeness strategies (25.0%) and the highest frequency of using positive politeness strategies (67.8%). Moroccan speakers are the second-lowest users of negative politeness strategies (31.6%). Turkish speakers employed negative politeness strategies (35.2%), which is the highest compared to other groups, and the least number of users of positive politeness strategies (53.1%) after natives. The results show the variations in using politeness strategies in various settings by L1 speakers of English and EFL learners.

The data analysis presented in Table 5, Table 6 and Table 7 offers a comprehensive understanding of the politeness strategies employed by L1 and EFL groups. 

Table 5 presents native speakers and EFL learners’ responses to WDCT scenarios as equal-status interlocutors.

Table 6 presents native speakers and EFL learners’ responses to WDCT scenarios as lower-status interlocutors.

Table 7 presents native speakers’ and EFL learners’ responses to WDCT scenarios as a stranger.

#### 4.2.1. Equal Status (Peers/Friends)—Scenarios 1, 5, 8

The findings reveal that British (native) speakers predominantly used negative politeness in peer contexts, relying on conventional indirectness, including hedging forms such as *“Would you mind…?” or “Do you mind if…?”* In this sample the negative strategy is dominant in L1 speakers’ responses in the peer context; (88.9%, *n* = 8/9) participants used these forms, often combining them with mild positive politeness to maintain rapport. On the other hand, EFL learners often used positive politeness (Congolese and Moroccans), with mild use of bald on-record by Turkish speakers. For instance, in scenario 1: *(British speaker; would you mind if I open the window? It is too hot in here). Expressions by EFL learners: (Turkish speaker; please open the window.), (Congolese speakers; Mate, please everyone, can I open the window?), (Moroccans; Please guys, can you open the window? It is too hot here)*.

For example: 

NS:

- *Would you mind if I open the window? (Softener) P3*
- *Do you mind if I could use some sunscreen? I forgot to bring mine. (Justification) P4*
- *Would you mind making some changes to the project before we submit it? (Softener) P6*

EFL learners, in contrast, relied heavily on positive politeness, using solidarity markers, friendliness, and direct modals such as “can”. They avoided imperatives but used more direct syntactic structures than the native group.

*TR:*

- *I forgot my sunscreen. Can I borrow yours? (Reason + approval) P31*
- *Please, open the window? (Softener + Directness) P6*

CG:

- *Do you guys mind if I open the window? (Inclusiveness) P15*
- *Excuse me my friend, please can I use some sunscreen of yours? (Attention getter & solidarity) P48*
- *Please, guys, may I have permission to make some changes in the project? (Initial softener) P45*

MC:

- *Sorry to bother you guys, can I open the window? (Apology) P30*
- *I forgot my sunscreen. Can I use yours? (Reason + request) P71*
- *Do you think it needs some changes? Let me do it and see if it’s ok? (Inclusivity) P16*
- *I appreciate your hard work, but can we make some changes? (Solidarity + appreciation) P30*

Across all L2 learner groups, positive politeness dominated, with frequent use of inclusivity (“we”), friendliness (“my friend,” “guys”), and apologies.

When the power is equal, British speakers used “*negative politeness*” and a slight use of positive politeness for the state of mind of the hearer and to preserve autonomy to make decisions independently. Eight out of nine speakers were found to use this strategy, e.g., “Would you mind…?” Conversely, EFL learners excessively used the linguistic marker “can” in the directive sense and in positive politeness strategies for asking permission. Participants were trying to use friendly expressions (“mate, my friend, please”) and to demonstrate inclusivity and solidarity. They avoided using direct expressions (imperatives) when producing permission speech acts (PSAs). These expressions show their struggle to be polite in their own way (culture). On the other hand, British speakers provide freedom of choice and try to keep a safe distance even from peers/friends.

#### 4.2.2. Lower Status (To Boss/Lecturer)—Scenarios 2, 3, 6

In interactions with higher-status interlocutors (boss or lecturer), both British speakers and EFL learners predominantly used *negative politeness*. Both groups show mutual awareness while communicating with high status (P+). The British group mostly uses formal and apologetic (*sorry to bother you, excuse me, would it be ok?, etc.*) linguistic markers (titles; e.g., *Mr. Smith*, compensation; *I’ll be right back*, urgency; *I’ll bring it right back*). Similarly, EFL learners were found to overuse “May I” in their strategies, which falls under *negative politeness* and is considered a polite expression towards high status in Brown and Levinson’s (1987) framework. In addition, non-native speakers of English often provide justification, stating the problem first and then seeking permission from a higher-status person (boss, lecturer). This indicates that EFL learners are trying to save face by being polite and softening the act of requesting permission in high social distance (Taguchi, 2006). In scenario 6 the permission-asking expressions of all groups are as follows: native speaker (*So sorry to interrupt, May I ask a question related to the topic?*), Turkish speakers *(May I ask a question, please?*), Congolese speakers (Excuse me, please, may I ask a question?), and Moroccans (*Sorry to interrupt, May I ask a question?*). These examples show that EFL learners and native speakers use negative politeness strategies in their own ways that reflect their recognition and realization of power in high social contexts.

NS:

- *May I leave a bit early today? I have a dental appointment. (formal permission + reason) P2*
- *I have a dental appointment, would it be ok if I leave early today? (reason + permission) P5*
- *So sorry to interrupt, may I ask a question related to the topic? (apologetic) P3*

TR:

- *I have a dental appointment tomorrow. May I leave early? (reason + permission) P31*
- *May I step out for a moment? (formal permission) P10*
- *With your permission, I need to step out for a while. (seeking approval) P44*

CG:

- *Please, I want to ask if I could leave early today. (seeking approval) P55*
- *Sir, may I leave early tomorrow? I have a dental appointment. (permission + reason) P55*
- *Please, Professor, I have a question. (initial “please”) P27*
- *Sorry, boss, I forgot to bring the print. Please let me get it quickly. (apology + reason) P15*
- *Sorry, it’s my bad, I forgot my prints, can I fetch them? (apology) P26*

MC:

- *Sorry, boss, it’s my bad. Can I step out to get a copy of my work? (apology + reason) P58*
- *Could I leave early for a dental appointment? I’ll make sure all my tasks are covered before I go. (compensation) P49*
- *Sorry for the interruption, I have a quick question about the topic. (apology) P49*

Learners frequently used reason + permission structures, apologies, and status labels (sir, boss, and teacher), demonstrating awareness of hierarchical norms.

Both groups, native and non-native participants, recognize the value of higher status equally by employing negative politeness when producing strategies for asking permission. To recognize the speakers’ authority, British participants used titles and formal addresses to make indirect requests for permission, e.g., “Mr. Keith or Professor Frank….” On the other hand, EFL learners avoided using titles and instead used “Sir,” “Madam,” and “Hocam” (teacher)”. British speakers frequently used “Would you mind…” and non-native speakers used the word “Permission” when asking for permission formally, avoiding directness in such situations. *Formal permission*, *attention getter, and apologetic markers are commonly used strategies across* the groups. 

British speakers used occupational titles (Mr. Keith, Professor Frank), while EFL learners used general honorifics (sir, madam, hocam). Learners also used explicit references to “permission,” e.g., “May I have permission…?”

#### 4.2.3. To a Stranger (Library/Park Warden)—Scenarios 4, 7

In the case of encountering a situation where the rank of imposition is high and the speaker is addressing a stranger (D+), the British group tends to produce *negative politeness* by using apologetic markers due to disturbance acknowledgement and assuring that strangers do not feel forced to comply (*“Sorry to bother you”, “sorry to disturb you” and excessive use of “Excuse me”*). Turkish, Congolese and Moroccans showed *positive politeness* (*“Could you help me?”, “Is it possible to sit here?”*), and EFL learners frequently used the directive *marker “Can”* in their strategies (“Can you help me? “Can I borrow your charger?”, “Can you lend me your charger?”) of permission speech acts. 

EFL learners often use the pronoun “My friend” when communicating with strangers to convey friendliness and a gentle demeanor. In contrast, British speakers typically use “Excuse me” to maintain a sense of distance, avoid closeness with a stranger, and show respect for the other person’s freedom and autonomy in making their own decisions. In scenario 7 all the groups’ expressions are as follows: British speakers (*I am so sorry to disturb you but I forgot my charger. Would it be possible to borrow yours for a couple of minutes?*), Turkish (*Excuse me, can I get your charger please?*), Congolese (*Please, can I borrow your charger?*), and Moroccans (*Sorry bro, can I use your charger?*). These responses (expressions) represent the difference in the use of strategy when a speaker is a stranger. Native speakers apologize for bothering a stranger and maintain their distance from the stranger, while EFL learners use “can” more often with the linguistic markers please and sorry. 

NS:

- *Excuse me, sir, is sitting on the grass allowed here? (Attention getter & honorific) P7*
- *Sorry, is it ok if I sit on the grass? (Apologetic) P8*
- *So sorry to bother you, but I just realized my laptop’s battery is low. Could I use your charger if you are not using it? (Autonomy preservation) P3*

TR:

- *Excuse me, my laptop is about to die, can I borrow a charger for a few minutes? (Attention getter) P12*
- *Excuse me, can I sit on the grass? (Attention getter) P16*

CG:

- *I appreciate your understanding. Can I sit on the grass? (Appreciation) P2*
- *Sorry to interrupt you, my battery is going to die. Can I borrow your charger for a few minutes, please? (Apology + reason + please) P51*

MC:

- *I appreciate your understanding. (Appreciation) P46*
- *Excuse me, sir, is it possible to sit here? (Attention getter + recognizing status) P32*
- *My laptop is about to die. Could I use your charger? (Reason + permission)*
- *It’s a beautiful day. Can I sit here for a while? (Solidarity) P19*

Learners occasionally used friendliness markers (“my friend”) with strangers, which differs from British norms, to reduce the intensity of the situation; the frequent use of “quickly,” “just,” “a bit,” and “a second” was found to be a cultural norm in British culture, framed as minimal disturbance. Native speakers tried to minimize the imposition by using excuse me and apologetic expressions.

EFL learners often use apologies, softeners like “please,” and openers to minimize imposition when making requests. They typically use the word “can” to ask for permission. In contrast, non-native participants frequently use “we” statements in scenarios involving changes to a project. They also tend to provide justifications or reasons when requesting permission from someone in a higher position. 

British participants, on the other hand, often use “I” as a personal pronoun, make frequent apologies, acknowledge the hearer’s need for autonomy (freedom from distraction), and avoid being direct when asking for permission. They employ “I” forms and hedges to help preserve the hearer’s independence.

### 4.3. Politeness and Social Harmony

The study investigates how the production of speech act (face-saving) strategies helps in understanding learners’ politeness levels and their social relationships within the cultures. 

NS:

- *I have a few minor tweaks to make. Do you mind if I make them* via *tracked changes so you can see what I’ve done? (Solidarity) P9*
- *Would it be ok if I left a bit early to go to a dentist appointment please? I can make up my time during the week. (Compensatory offer) P6*
- *Hi, I am really sorry to bother you, but my laptop battery is about to die, and I noticed you have the same charger. Would it be okay if I borrowed it for just a few minutes? I’d really appreciate it, would be a huge help! (Appreciation marker) P7*

TR:

- *My dear friend, I forgot to bring sunscreen. Can I borrow yours? (Solidarity) P1*
- *I’m sorry, but I couldn’t manage to get the copies. If you allow me, can I take the printouts and come right away? (Self-blame) P65*
- *How about checking it one last time and sending it? (Indirect suggestion) P44*

CG:

- *My friend, I have some good ideas about our project, and I want to present them to you. We will decide together. (Solidarity) P15*
- *Please sir, can I have your permission to leave early? I have a dental appointment. (Please* as a *formulaic softener) P45*
- *Apologies for the interruption, I forgot to bring a print copy of my work. May I step out quickly to print it so I can present it during the meeting? (Apology) P59*

MC:

- *Sorry boss it’s my bad, can I step out to get the copy of my work? (Self-blame) P58*
- *I appreciate your hard work in the project but can I make some changes.” (Appreciation) P30*
- *I’ve been thinking about a few adjustments that might improve our project before we submit it. (Offer) P49*
- *Can we go over them together to see, what do you think? (Solidarity) P49*

Employing politeness can help both the addresser and the addressee avoid misunderstandings and uncomfortable situations, protect others’ rights and freedoms, and emphasize social harmony (Afriana et al., 2025). The findings of the study show that both native speakers (NS) and non-native speakers (NNS) are trying to save their faces and use different strategies to produce PSAs. L2 speakers of English preferred (*solidarity, transparency offer, compensatory offer and appreciation*) in their use of strategies to maintain social harmony. L2 speakers’ strategies are (solidarity, self-blame, indirect appreciation, and apology). Both groups show common ground to some extent in their use of politeness strategies such as *solidarity* and *appreciation*. The results indicate that EFL learners used inclusive/solidarity strategies and excessive use of “please” and “sorry” to be appreciated in social contexts. On the other hand, consistent, dominant-strategy negative politeness was employed by British speakers to respect hearers’ freedom of action, maintain respectful behavior, and avoid closeness with strangers and with their friends/classmates.

When it comes to asking for permission from an authority figure (boss or lecturer), both groups show common ground in their use of politeness strategies. The results show that both groups, L1 and L2 speakers, employed negative politeness strategies to avoid the risk of losing their faces while communicating to an authority figure, indicating awareness of social and cultural awareness in a specific context. However, the differences in the use of politeness strategies in other contexts can lead to disagreements. These interactive choices of strategies might indicate cultural differences and L1 transfer, leading to misunderstandings between L1 and L2 speakers of English. Although EFL learners show differences in the use of strategies, their polite intentions are reflected in the use of linguistic markers (please and sorry). However, these differences can lead to misunderstanding due to excessive use of “can” and demanding expressions such as “allow me to sit on the grass.” To avoid misjudgments and misinterpretations, L2 speakers of English require intercultural training and socio-pragmatic awareness to mitigate face-threatening situations.

To maintain social relationships, it is crucial to educate learners not only in the use of vocabulary and grammar but also in the awareness of social and cultural norms of the target language by implementing explicit/implicit instructions. The key to maintaining a successful relationship in cultures is social harmony. To maintain this unity, politeness strategies are linguistic and behavioral tools to avoid conflicts and preserve personal relationships. It is a step towards politeness, which involves showing courtesy and kindness by acknowledging mutual respect and boundaries towards others and considering their social norms. It is a fundamental aspect of verbal and non-verbal strategies across cultures (Leech & Tatiana, 2014). Politeness in communication helps individuals understand and interact with one another across various cultures and social settings. Effective and favorable communication is an indicator of social harmony and improved human relationships (Afriana et al., 2025).

## 5. Discussion

The findings demonstrate that both British speakers and EFL learners orient toward maintaining social harmony, though they do so through different politeness strategies shaped by cultural norms, linguistic resources, and socio-pragmatic awareness. British speakers employed negative politeness at high rates, demonstrating consistent preferences for autonomy-preserving politeness relative to EFL learners. Their use of “Would you mind…?” aligns with previous research showing that British English favors indirectness to preserve the hearer’s freedom of action. Culpeper and Demmen (2011) demonstrated that negative politeness is the most frequently employed strategy among native speakers of English, where speakers consistently use hedges foregrounding the hearers’ autonomy in line with negative face concerns. Aba Poulos and Smits (2023) indicated native speakers’ use of negative politeness strategy in communicating with peers and low-imposition situations. To maintain social harmony and balance respect for others, native speakers maintained distance through apologetic openers and hedges to keep a safe distance from the interlocutor, particularly strangers. EFL learners, however, relied on positive politeness, using solidarity markers, friendliness, and inclusive pronouns. This aligns with findings by Mukharomah and Sumanto (2023), who note that positive politeness is common in peer interactions in many cultures. Turkish and Congolese learners used positive politeness, while Moroccan learners occasionally used more direct strategies. Şanal and Ortaçtepe (2019) and Marti (2006) found that Turkish EFL learners’ pragma-linguistic variations lead to the use of bald on-record strategies, which is caused by Turkish L1 transfer. According to Nwoye (1992), Congolese use of positive politeness strategies and kin-based addresses forms oriented toward solidarity and communal belonging are the normative mode of peer interaction. Alaoui (2011) suggested that Moroccans’ use of positive politeness strategies is culturally embedded mechanics to deal with face-threatening acts and the use of solidarity-driven forms (my friend, everyone, mate).

In higher-status interactions, both groups converged on negative politeness, demonstrating some shared awareness of hierarchical norms. Learners’ use of apologies, formal modals, and reason-giving structures suggests the development of socio-pragmatic awareness. This supports Yaghoubzadeh (2021), who found that speakers in hierarchical relationships prioritize deference mechanisms to mitigate face-threatening acts. L1 (British) speakers used “could/would” to create distance, while learners used “may” in formal contexts and can in peer interactions. These differences reflect pragma-linguistic transfer in communication rather than the intention to be rude or disrespectful towards others (Taguchi, 2006). L. F. Kasper (1997) emphasized the relevance of sociocultural context in human interactions and education, while Langford (1938) suggested that pragmatics examine meanings as perceived by interlocutors, not their intentions to be impolite. Although EFL learners are sometimes perceived as impolite due to linguistic limitations (Alisoy, 2025), this study shows that learners actively attempt to mitigate face-threatening acts by using various linguistic softeners (please), attention and markers. These choices can lead to misunderstanding and misinterpretation by the native speakers, and they can be perceived as demanding or overemphasizing rather than asking permission politely. These observed differences in the production of politeness strategies across groups reflect cultural variations in politeness norms, though the causes could be specific cultural factors (L1 transfer, exposure to target language, educational background) that would require additional analysis for future studies. Munir and Yavuz (2024) emphasized pragmatic knowledge in education that can enhance students’ receptive and productive skills, arguing that these skills elevate learners’ socio-pragmatic awareness and communication. The findings highlight the need for intercultural education and training, explicit/implicit instructions in socio-pragmatic norms, pragma-linguistic forms, and cross-cultural variations. As Ross and Kasper (2013) suggest, exposure to diverse communicative norms enhances learners’ pragmatic development and intercultural awareness while interacting in various contexts.

## 6. Conclusions

This study offers a comprehensive analysis of cross-cultural behavioral strategies in permission speech acts among L1 and L2 learners with Turkish, Congolese, and Moroccan backgrounds. The findings revealed divergent behavioral orientations between the groups across various contexts. L1 (British) speakers typically prefer negative politeness strategies, which emphasize distant differences in interpersonal interactions and provide the hearer with a choice of freedom. Conversely, EFL learners tend to favor solidarity-based strategies (positive politeness), prioritizing relational association and social cohesion even when significant social distance exists (to strangers) and interlocutors are viewed as equals. This orientation suggests socially established tendencies toward sociocultural variations rather than recognizing formal (social) status; their strategy development pattern reflects collective values that emphasize social collectivisms over personal independence.

Critically, both L1 speakers and EFL learners converge on the use of negative politeness strategies, indicating a shared recognition of hierarchical contexts and reflecting awareness of the social power dynamics at play. This tendency suggests that cultural values, perhaps, shape baseline behavioral choices, with contextual power dynamics considered a universal modulating effect on interpersonal conduct.

The observed divergence carries meaningful implications for intergroup relations in pluralistic societies. It is an essential aspect of the learners’ behavioral choices that could be influenced by their first languages, which manifests as L1 transfer and unfamiliarity with intercultural social standards. When individuals from various linguistic and cultural backgrounds interact, misunderstanding arises where one group of individuals expects solidarity and the other distance. Such misalignments can lead to misunderstandings and misinterpretations. These findings highlight the need for a diversity framework that helps interlocutors of multicultural norms to actively unite diverse social identities and legitimize diverse behavioral accumulations. In educational and organizational settings, developing linguistic resources and entrenched awareness of culturally grounded behavioral variations may reduce intergroup friction and enhance social integration. Rather than requiring minority groups to abandon their culturally embedded strategies, the learners’ use of apologies, (*sorry to bother you, may I ask a question?*), attention getters (*Excuse me, my laptop is about to die, can I borrow a charger for a few minutes?*), softeners (*please*), and justifications (*It is ok if I open the window for a while? It feels a bit warm and stuffy here*) suggests attention to face-saving/threat mitigation, which showcases their understanding of social norms and their commitment to maintaining social harmony in diverse communicative situations. Although learners reflect harmony to some extent, to achieve pragmatic and standard communicative skills, explicit/implicit pedagogical implications can enhance their use of language in various settings. These findings suggest that EFL pedagogy might benefit from awareness-raising regarding British English and cultural norms, explicit instructions across power/distance contexts and cross-cultural pragmatic training recognizing that EFL learners’ strategy variations reflect cultural norms rather than incompetence. This exploratory study highlights the importance of designing culturally inclusive curriculum activities that accommodate behavioral pluralism and ultimately contribute to a nuanced understanding of cross-cultural interactions.

Through this exploration, EFL learners employ distinct politeness strategies reflecting their cultural backgrounds and L1 influences; the study supports an intercultural competence model that values linguistic and pragmatic pluralism over native speaker conformity. The study highlights the importance of designing culturally inclusive curriculum activities that accommodate behavioral pluralism and ultimately contribute to a nuanced understanding of cross-cultural interactions.

### Limitations and Future Research

This exploratory study has several considerable limitations. First, the insufficient sample size (*n* = 9) of native speakers limits the generalizability and is insufficient for establishing definitive “norms” of British English permission speech acts (PSAs). However, these speakers have provided valuable context for understanding the differentiation between English L1 speakers’ and L2 speakers’ speech act production patterns observed in this small sample. Consequently, the findings regarding British speakers should be considered as patterns observed rather than as characteristics of British English speakers. The EFL learners’ sample size (*n* = 54) represents first-year university students at one educational institution in North Cyprus; the findings might not generalize to other proficiency levels, exposure to English, geographical locations, or educational contexts. All groups shifted toward negative politeness in high-status contexts, suggesting contextual awareness. However, whether these choices successfully maintain social harmony or would be perceived as polite by native interlocutors remains unknown without perception studies—an important limitation acknowledged as future research.

Additionally, the study did not systematically examine the role of formulaic expressions in observed PSA patterns in British English teaching versus cultural pragmatic norms. Different EFL curricula could account for documented variations in strategies. Social class and background were also not investigated as variables; future research could focus on directness/indirectness in politeness that may correlate with learners’ socioeconomic status independently of language and culture. The written discourse completion task (WDCT) methodology elicits controlled written responses, rather than spontaneous interactive conversations. Real-time conversations might affect learners’ permission-asking strategies and politeness choices. While EFL learners employ softening strategies (please, apology, and solidarity markers), over-reliance on model-based requests (Can I…?) and less use of hedging and conditionals, as reflected in L1 speakers’ patterns, might be evaluated in perception-based research. This remains an empirical question requiring perception validation.

## Figures and Tables

**Table 1:** Situational variables.

*Scenarios*	*Contexts*	*Power (P)*	*Distance (D)*	*Imposition (R)*
Item 1	Open a window	Equal =	Low	Low
Item 2	Leave work early	Higher +	High	High
Item 3	Step out of the meeting	Higher +	High	High
Item 4	Sit on the grass	Higher +	High	Low
Item 5	Borrow sunscreen	Equal =	Low	Moderate
Item 6	Ask question (lecturer)	Higher +	High	Low
Item 7	Borrowing a charger	Equal =	High	High
Item 8	Changes in project	Equal =	Low	Moderate

Note: In social contexts “=” represents equal status and “+” represents higher status.

**Table 2:** Coding taxonomy for permission speech acts (PSAs).

Strategy	Sub-Strategy	Tactical Device	Linguistic Example
Bald on-Record (BOR) Doing an act directly	- Direct imperative - Direct questions - Declarative statement - Direct modals (can/will)	Imperative verbs, tag questions, questions without softeners (please), direct announcement or action, modal verbs without qualifications	“Open the window.” “Lend me your charger.” “Can I open the window?” “I need to leave early.” “I want to make changes.” “Will you allow me?”
Positive Politeness (PP) Appeals to the hearer’s desire emphasizing common ground, warmth and friendship	- Solidarity/in-group markers - Justification/reasoning - Complements/appreciation - Inclusive language (We/Our) - Cooperative framing	Kinship terms, group identity, because clause, explanations, explicit praise, thanks, appreciation markers, “We”, “our”, “let’s”, suggestions, problem solving	“Mate”, “My friend, can I borrow your charger?” “Guys, can we make changes?” “My laptop battery is dying can I use your charger?” “I appreciate your hard work, but can we make changes?” “How about we check it one more time?” “Let’s make changes together.”
Negative Politeness (NP) Respect the hearer’s negative face, freedom from imposition	- Conditional interrogatives - Hedges/softeners - Apologies/regret expressions - Optionality markers - Indirect permission forms - Distance maintaining - Compensation/Commitment offers - Minimal imposition indicators	(Could/Would+ conditional structure), (please), (Minimizers: just, a bit, a few, only, quickly, a moment), (Sorry, excuse me, apologies, pardon), (If you don’t mind, if possible, if you allow), (May I, Might), (Mr., Ms., Professor, Sir, Madam), (I’ll be right back, I’ll make up the time), (Soon, quickly, just now, for a few seconds)	“Would you mind if I open the window?” “Could I possibly borrow your charger?” “Just a moment, can I ask a question?” “Could I use your charger for just a few minutes?” “Sorry to bother you, but can I ask a question?” “Apologies for interruption, may I step out?” “If you don’t mind could I open the window?” “May I ask a question?” “Professor Smith, would it be possible to leave early?” “Sir, may I have permission to leave early today?” “Could I leave just for a few minutes?”
Off-Record (OR) Communication through hints, hedges, ambiguities, letting the listener infer the meaning	- Hints/Embedded request - Rhetorical questions - Impersonal constructions - Indirect suggestions	Embedded conditionals, passive voice, existential “there is”, “What do you think about…?” “Would it help if…?”	“I notice you have a charger.” “My laptop battery is low.” “Is it allowed to sit on the grass?” “Might it be ok to make changes?” “What do you think does it need changes?” “Would it help if I fetch the print copies?”

**Table 3:** Participants’ demographics.

Demographics	British Natives (*n* = 9)	Turkish EFL (*n* = 16)	Congolese EFL (*n* = 19)	Moroccan EFL (*n* = 19)
Age	22–35	18–24	18–26	19–23
Gender	5M, 4F	8M, 8F	10M, 9F	9M, 10F
Years Studying English	L1 English speakers	Range: 5–15	Range: 3–12	Range: 5–15
Self-reported Proficiency Level	—	B1–B2 (11 B1, 5 B2)	B1–B2 (14 B1, 5 B2)	B1–B2 (13 B1, 6 B2)
Exposure to English	Native environment	Classroom + media (M = 5.2 h/week)	Classroom + media (M = 3.8 h/week)	Classroom + media (M = 5.0 h/week)
Time Abroad (English-speaking country)	Native environment	None	None	None

Note: L1 speakers (British), L2 speakers (EFL; Turkish, Congolese, Moroccans), M = mean score.

**Table 4:** Overall distribution of politeness strategies by group.

*Politeness Strategy*	British (*n* = 9, 72 Resp.)	Turkish (*n* = 16, 128 Resp.)	Congolese (*n* = 19, 152 Resp.)	Moroccan (*n* = 19, 152 Resp.)	Total (*n* = 63, 504 Resp.)
*Negative Politeness*	59 (81.9%)	45 (35.2%)	38 (25.0%)	48 (31.6%)	190 (37.7%)
*Positive Politeness*	12 (16.7%)	68 (53.1%)	103 (67.8%)	96 (63.2%)	279 (55.4%)
*Bald on-Record*	1 (1.4%)	15 (11.7%)	12 (7.9%)	8 (5.3%)	36 (7.1%)
*Off-Record*	0 (0.0%)	0 (0.0%)	1 (0.7%)	0 (0.0%)	1 (0.2%)
*TOTAL*	72	128	152	152	504

*n* = total number of participants and total number of strategies.

**Table 5:** Equal status (peers/friends)—scenarios 1, 5, 8.

Native/EFL Learners	Strategy	Functions	Linguistic Markers & Examples
Native Speakers	Positive politeness/ negative politeness (NP)/(PP)	Solidarity/Inclusivity Conventional Indirectness (autonomy preservation) Reason + permission OR permission + reason	*“Just a bit, is it ok? Would you mind? Would it be ok?”* *e.g., Would you mind if…* *e.g., Is it ok, if I open the window, we need some fresh air in here?* *e.g., I haven’t put any sunscreen on, do you have any?* ***OR*** *e.g., Do you mind if I could use some sunscreen? I forgot mine.*
Turkish Speakers	Positive politeness/Bald on-record (PP)/(BOR)	Solidarity, friendly grounding, inclusivity, seeking approval, (direct statement + permission)	*My friend, mate, together, can I, can you,* etc. *if it is ok for you?* *e.g.,* *If you want, we can change our project* *I want to make changes.* *I wanted to get your opinion…* *I forgot my sunscreen. Can I borrow yours?*
Congolese Speakers	Positive politeness (PP)	Lexical softening, social bonds	*Use the “Please”, I was wondering,* *Bro, mate, friend, we,* *e.g., We will decide together for some changes.* *Would you help me?*
Moroccans Speakers	Positive politeness (PP)	Solidarity, Complement, appreciation, apology, Consultation	*Sorry to bother you guys, can I open the window?* *Appreciate your hard work,* *e.g., Sorry, but I forgot my sunscreen at home.* *Excuse guys, can we open the window?* *What do you think?*

**Table 6:** Lower status (to boss/lecturer)—scenarios 2, 3, 6.

Native/EFL Learners	Strategy	Functions	Linguistic Markers & Examples
Native Speakers	Negative politeness (NP)	Compensation, admitting mistakes, Excuse for disruption (apology) self-correction (repay the favor) Reason + permission OR permission + reason	*Quickly, if I nip out, sorry, excuse me,* *I’ll be right back, Mr. xxx, silly me.* *e.g., excuse me for a few seconds…* *Sorry, if you could excuse me…* *e.g., I have forgotten my notes, could you excuse me to fetch them? OR* *e.g., May I quickly step out to get my work from the office?*
Turkish Speakers	Negative politeness (NP)	Apologetic, Seeking approval, Informative, formal permission, directness, Assumption of permission	*Sorry, I forgot to print.* *With your permission,* *May I ask a question?* *I have a question about…* *I won’t be able to come.* *e.g., With your permission, can I ask you a question?* *Sorry, can I go for a dental appointment?* *Sorry, can I change some things?* *With your permission, can I leave early?*
Congolese Speakers	Negative politeness (NP)	Initial softener repetitive, explicit permission, apologetic interruption, status labeling,	*Please, sir, can I have a day off, please?* *I want to ask your permission; I want to go out,* *I want to know if …,* *Sorry to interrupt you…,* *Boss, sir, Teacher,* *e.g., I want to know if I can leave early.*
Moroccan Speakers	Negative politeness (NP)	Compensation, Excuse for disruption (apology) self-correction (repay the favor) Reason + permission OR permission + reason	*e.g., I’ll make sure all my tasks are covered before I go.* *I am really sorry to bother you.* *e.g., Excuse me sir, I could not bring my printed copy of my work, can I step out to get it?*

**Table 7:** To a stranger (library/park warden)—scenarios 4, 7.

Native/EFL Learners	Strategy	Functions	Linguistic Markers & Examples
Native Speakers	Negative politeness (NP)	Past-tense hedge, rule inquiry Apologetic openers, Attention getter,	*Excuse me, by any chance, is it allowed? Can I, could I borrow, a huge help, sorry to bother you.* *e.g., Is it allowed to sit on the grass?* *Sorry, is it ok if I sit down on the grass?* *Excuse me, sorry to bother you…*
Turkish Speakers	Positive politeness (PP)	Attention getter, status recognition,	*Excuse me, can I sit here?* *Can I borrow it? “If you allow me…* *Can I take permission to sit here?*
Congolese Speakers	Positive politeness (PP)	Status recognition, attention getter, appreciation	*I want to ask if I can sit in the park.* *I want to ask for your charger for a few minutes.* *e.g., I appreciate your understanding.*
Moroccan Speakers	Positive politeness (PP)	Attention getter, status recognition, appreciation	*Excuse me sir, is it possible to sit here?* *e.g., My laptop is about to die, could I use your charger?*

## Data Availability

The data presented in this study are available on request from the corresponding author.
